# Treatment of parvovirus B19 viremia to facilitate kidney transplantation in a patient with collapsing glomerulopathy 

**DOI:** 10.5414/CNCS110113

**Published:** 2020-05-29

**Authors:** Vinay Nair, Nicholas Jandovitz, Kenar D. Jhaveri, David Hirschwerk, Elliot Grodstein, Vanesa Bijol, Ernesto Molmenti, Lewis Teperman

**Affiliations:** 1 Department of Medicine, Donald and Barbara Zucker School of Medicine at Hofstra/Northwell,; 2 Department of Pharmacy, North Shore University Hospital, Northwell Health,; 3 Department of Surgery, and; 4 Department of Pathology, Donald and Barbara Zucker School of Medicine at Hofstra/Northwell, Manhasset, NY, USA

**Keywords:** kidney transplantation, parvovirus B19, collapsing glomerulopathy, cidofovir

## Abstract

Collapsing glomerulopathy (CG) is a severe form of glomerulopathy which results in nephrotic syndrome and often ensues in rapid progression to end-stage kidney disease (ESKD). Although most commonly a result of HIV infection, other conditions such as parvovirus B19 (PB19) infection have been associated with CG. We present a case of an 18-year-old male with CG associated with PB19 infection who was heterozygous for *APOL1* G1 and G2 genetic variants. In an attempt to treat, he was started on intravenous immunoglobulin (IVIg), however rapidly progressed to ESKD. During workup for a living donor kidney transplant he was found to have persistent low-grade PB19 viremia. Despite having no major immunodeficiency and given subsequent courses of IVIg, viremia continued to persist. In a final attempt to eradicate the PB19 we began treatment with cidofovir, an antiviral agent with in vitro efficacy against PB19. Subsequent to initiation of cidofovir, PB19 viremia slowly cleared after which he received a living unrelated kidney transplant. The patient had an early cellular rejection treated with rabbit antithymocyte globulin after which he recovered kidney function without signs of recurrent CG. Our case report suggests efficacy of IVIg and cidofovir for persistent PB19 infection in ESKD to allow subsequent transplantation, while minimizing the risk of recurrent CG.

## Introduction 

Collapsing glomerulopathy (CG) is a rapidly progressing glomerular disease that often is resistant to treatment. Classically, CG occurs in association with human immunodeficiency virus (HIV) infection. HIV-induced CG is thought to be triggered by infection of kidney epithelial cells by HIV in genetically susceptible hosts [1, 2]. Genetic susceptibility has been suggested by the strong association with African ancestry and, more specifically, single nucleotide polymorphisms in the *APOL1* gene (G1/G1, G1/G2, or G2/G2) [2]. Parvovirus B19 (PB19) is a virus that has also been associated with CG [3]. Infection has been shown to coincide with the onset of kidney disease, and PB19 has been found in epithelial cells of patients with CG [4]. The exact mechanism by which PB19 infection leads to CG, however, is unknown. Although our understanding of CG has expanded, treatment options remain poor. Treatment of HIV-induced CG revolves around antiretroviral therapy, while in idiopathic CG, steroids and immunosuppressive agents are often used to no avail. As studies suggest treatment with antiretroviral therapy improves renal survival, it is intuitive that treatment of PB19-associated CG may also improve renal outcome [5]. 

While data is limited to case reports and case series, in PB19-induced CG, kidney transplantation can be performed, however recurrent disease may occur. Whether recurrence depends upon PB19 status of the recipient, genetic background of the donor, or is independent of these factors is unknown. We present a patient diagnosed with PB19-associated CG who rapidly progressed to end-stage kidney disease (ESKD) despite treatment with intravenous immunoglobulin (IVIg). Further evaluation was negative for significant immunodeficiency but positive for *APOL1* genetic variants G1 and G2. Subsequently, he was treated with cidofovir which eventually resulted in PB19 viral clearance and a successful live donor kidney transplant. Our case suggests efficacy of IVIg and cidofovir for persistent PB19 infection in ESKD to allow subsequent transplantation, while minimizing the risk of recurrent CG. 

## Case report 

**Figure 1:**
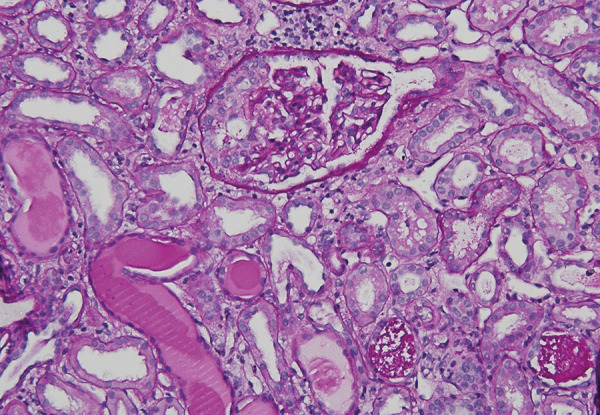
The glomerulus reveals features of collapsing glomerulopathy, with epithelial cell proliferation and collapse of underlying capillary walls. Tubules reveal flattening of the epithelium, distension of lumens, and focal intraluminal accumulation of cellular debris (periodic acid-Schiff stain, × 200).

The patient is an 18-year-old African American male hospitalized for elevated serum creatinine (2.74 mg/dL) and nephrotic syndrome. Serological evaluation revealed negative anti-nuclear antibody, anti-dsDNA, c-ANCA, p-ANCA, hepatitis B and C antibodies, HIV, and anti-glomerular basement membrane antibody. Complement C3 and C4 levels were normal, and serum free light chain ratio was not elevated or suppressed. Kidney biopsy revealed morphological findings of CG (Figure 1). PB19 viral load was elevated at 96,600 IU/mL. There were no recent respiratory infections or history of recurrent infections as a child. He was given 2 g/kg IVIg in an attempt to treat the PB19. His initial viral load increased to 107,000 IU/mL but then subsequently decreased to 800 IU/mL by November 2017. Despite the reduction in PB19 titer, his kidney function rapidly decreased and he was initiated on dialysis. He began evaluation for kidney transplantation, and a non-related African American living donor was identified. 

**Figure 2:**
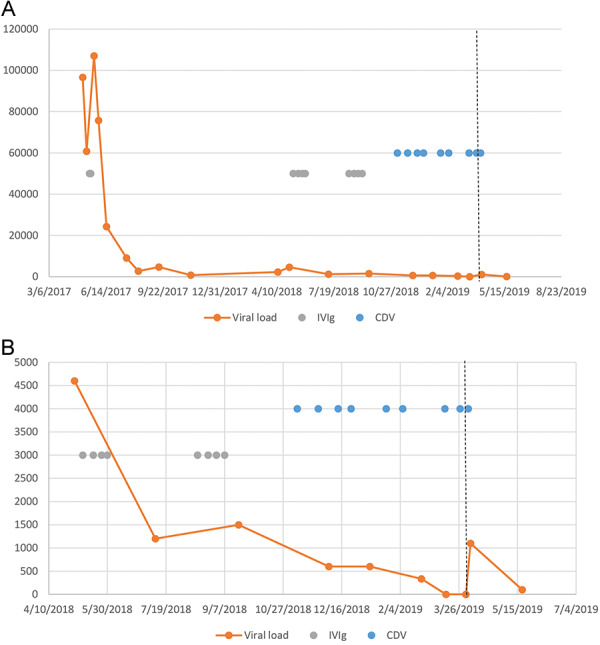
Kinetics of PB19 viremia from detection to 6 weeks after transplantation (A) and a magnified view of viral loads of PB19 (notice the Y axis difference) from the initiation of the second course of intravenous immunoglobulin (B). IVIg = intravenous immunoglobulin 500 mg/kg (gray dot); CDV = cidofovir 0.5 mg/kg (blue dot); dotted line = day of transplantation.

In an attempt to eradicate the PB19 infection before transplantation he was given 2 more courses of 2 g/kg IVIg over the next 4 months. His viral load remained positive at low levels. Further immunologic evaluation was negative except for low mannan-binding lectin (< 70 ng/mL, normal > 100). PB19 IgG was positive, but PB19 IgM was negative. Genetic testing found the patient to be heterozygous for *APOL1* G1/G2 alleles. A second attempt to eradicate the PB19 viremia began with cidofovir treatment based on published in vitro efficacy [6]. The patient received cidofovir 0.5 mg/kg every 2 weeks as suggested by a pharmacologic study of cidofovir in patients with kidney disease [7]. He received a total of 7 doses of cidofovir before PB19 viral load became negative. As his donor was available for a limited window of time, it was decided to proceed with living donor transplantation after administering the 8^th^ dose of cidofovir. Five days after the 8^th^ dose of cidofovir, he received a living unrelated kidney transplant. Induction immunosuppression included methylprednisolone 500 mg at the time of surgery and basiliximab on post-operative day (POD) 0 and 4. He was maintained on tacrolimus, mycophenolate mofetil, and corticosteroids were tapered to prednisone 5 mg daily over 5 days. Due to the intense immunosuppression associated with induction, the patient was given a 9^th^ dose of cidofovir on post-operative day 2 (7 days from the previous dose of cidofovir). Creatinine immediately decreased following transplant but then increased by POD 4. PB19 viral load was repeated and found to be positive at 1,100 IU/mL. Subsequently, a kidney biopsy was performed on POD 5 which revealed a Banff grade 2a rejection. As there was no evidence of CG on biopsy but clear vascular rejection, the clinical team decided that the risk of worsening PB19 viral replication by aggressive treatment of rejection was outweighed by the clear negative impact of ongoing rejection. Therefore the patient was treated with 10 mg/kg of rabbit antithymocyte globulin and steroid bolus. Kidney function improved, and a repeat kidney biopsy 2 weeks after treatment revealed mild acute tubular injury and no evidence of CG or podocyte effacement. Repeat PB19 viral load 1 month later revealed a decrease in PCR to the lower limit of laboratory testing < 199 IU/mL. The kinetics of PB19 viremia from the time of diagnosis to 6 weeks after transplantation is seen in Figure 2. Other viruses including cytomegalovirus and polyoma (BK) virus were not detected. Ten months post transplant his creatinine is 2 mg/dL, and there is no proteinuria. PB19 has remained detected but < 199 IU/mL up to 6 months post transplant after which it has no longer been tested. 

## Discussion 

An increasing body of literature suggests an association between PB19 infection and CG. In patients with persistent parvoviral infection that progress to ESKD, not much is known about the risk of recurrence after kidney transplantation. We offer a novel in-vivo treatment option for persistent PB19 infection and report on post-transplant outcome. 

Several studies have found high prevalence of PB19 DNA in patients with CG [4]. Whether due to direct infection of epithelial cells or indirect effects of viral infection is unclear. In a study by Moudgil et al. [4], PB19 DNA was detected in 78% of kidney biopsies from patients with CG. Viral genome was detected in the renal epithelial cells suggesting a direct effect of viral infection on cell hyperplasia. A similar study found PB19 DNA in 90% of patients with CG [8]. Since the discovery of *APOL1* it is now clear that individuals with at-risk genetic variants (G1/G1, G1/G2, or G2/G2) carry a much higher risk of CG when also infected by HIV [2]. Similarly, it is likely that there is an interplay between PB19 and *APOL1* which leads to CG. A report by Besse et al. [9], details the development of CG over 3 biopsies in a patients with *APOL1* high-risk alleles and acute PB19 infection. Our patient was a healthy African American male who developed CG in a similar manner, in the setting of high-titer PB19 viremia. 

Treatment of CG can be difficult. Before the advent of HIV antiretroviral therapy, CG was the most common glomerulopathy in HIV-positive individuals. The advent of antiretroviral therapy has lowered the incidence of CG suggesting a role of treatment by viral control. If we hypothesize that a similar process exists between PB19 and *APOL1*, it is reasonable to believe that treatment of PVB19 may improve kidney outcomes in PB19-associated CG. Unfortunately, treatment options for PB19 are limited. IVIg has been used after kidney transplantation to treat PB19-induced aplastic anemia [10]. However, few authors have reported on the use of IVIg for chronic PB19 infections in patients without immunodeficiency. In one report, PB19-associated vasculitis and viremia improved after IVIg [11]. Both cidofovir and brincidofovir have been found to inhibit PB19 replication in vitro. Despite the inhibitory properties of cidofovir on PB19 replication in vitro, to our knowledge it has never been used in vivo and is generally contraindicated in kidney disease [6]. Brincidofovir, a lipid conjugate of cidofovir, seems to be safe in chronic kidney disease (CKD) however is not FDA approved [12]. Although avoided in CKD, pharmacokinetics of cidofovir have been studied in hemodialysis patients [7]. Based on this study, we dosed our patient with cidofovir 0.5 mg/kg every 2 weeks. Although viremia was low, it began to increase after IVIg was completed but cleared after 7 doses of cidofovir. He was given an 8^th^ dose of cidofovir in preparation of transplant, then given a 9^th^ dose post transplantation during the period of intense immunosuppression. Our patient did not exhibit any side effects of treatment. This is an important finding as there are no published reports on the successful use of cidofovir in vivo to treat PB19 viremia and limited data on its use in ESKD. However, the decision to start cidofovir cannot be taken lightly as cidofovir has significant nephrotoxicity and may hasten ESKD in patients with CKD. Our patient had a transient increase of PB19 viremia post transplant which quickly returned to the lower limit of laboratory testing despite receiving antithymocyte globulin for rejection. 

To our knowledge there is only one study which helps understand the risk of recurrent PVB19-induced CG after transplantation. Barsoum et al. [13] reported on a patient who developed anemia and nephrotic proteinuria which culminated in graft failure of her second kidney transplant. After being transplanted for the third time, the anemia and proteinuria reoccurred and biopsy revealed CG. The patient was found to have PVB19 viremia and lost the graft. Retrospective analysis of stored blood from the second transplant revealed PVB19 viremia to also have been present. Finally, after withdrawing all immunosuppression and clearing PVB19 viremia, she received a fourth transplant without recurrence of anemia or CG, suggesting PVB19 should be cleared before retransplantation. However recent studies on de novo CG suggest that it is the donors *APOL1* genotype which actually dictates the risk for CG [14, 15]. In one such study, 5 transplanted kidneys that went on to develop CG in different recipients were all found to carry two *APOL1* risk alleles. Four of the 5 patients that received these kidneys had viremia (CMV and BK) preceding the development of CG; suggesting a “second hit” may trigger CG in a donor organ which exhibits high-risk alleles [14]. We could have sent our donor’s *APOL1* genotype, however it was not performed as it was not a part of our donor workup process and PB19 treatment was successful. 

In conclusion, PB19 infection in patients with high-risk *APOL1* alleles are at risk of developing CG. In such patients with chronic viremia, there are no proven treatment options. Our case suggests IVIg and cidofovir may be helpful in treating chronic PB19 viremia. Whether earlier eradication of viremia can alter the course of CG is unknown. Furthermore, in such patients where viremia resolves, kidney transplantation appears to be a safe option. Whether this is a result of control of viremia, thereby avoiding a “second hit,” or the donors *APOL1* genotype, or possibly both requires further study. 

## Funding 

There was no funding in creation of this article. 

## Conflict of interest 

KDJ serves as a consultant for Astex Pharmaceuticals. The other authors have no conflict of interest to declare.
